# Necrotizing Enterocolitis in Full Term Neonates: Is There Always an Underlying Cause?

**Published:** 2013-07-01

**Authors:** Olivier Abbo, Luke Harper, Jean-Luc Michel, Dushka Ramful, Audrey Breden, Frederique Sauvat

**Affiliations:** CHU Reunion, Saint Denis, Reunion

**Keywords:** Enterocolitis, Full term, Pediatric surgery

## Abstract

Objective: To review our experience with full-term neonates with necrotizing enterocolitis (NEC) and to compare its characteristics to those published in the literature.

Design: Retrospective review of all neonates born after 35 weeks of gestation managed in Reunion Island for NEC from 2000 to 2012.

Results: Among the 217 diagnosed NEC, 27 patients (12.4%) were full term neonates, who were born at a mean gestational age of 36.8 ±1.7 weeks. The mean onset of the disease was 12.1±11.2 days after birth. Twenty patients had underlying causes (15 organic pathologies of the child, 3 isolated maternal disease, and 2 infections); 7 had idiopathic NEC. Surgery was required in 12 patients (37.5%) at 23.2±20 days after birth. NEC affected most of the time the colon (n=6) and the rectum (n=3). Overall survival rate was 88.8% (24/27). Two patients required partial non-enteral nutrition for1.3 and 2.1 years.

Conclusions: NEC in full term neonates is a rare pathology. The onset of the disease in our experience was slightly later than described in the literature, but remains earlier than in the premature population. In some cases, no obvious cause can be found, suggesting a different pathogenesis. Further investigations are required in order to better understand this pathology. The goal will be to find measures to reduce global mortality.

## INTRODUCTION

Necrotising enterocolitis (NEC) remains a major life threatening disease during the neonatal period. Despite the improvements in management of these patients, the associated morbidity and mortality remains high, with an average reported death rate of 20% [1, 2]. The development of efficient strategies for the management of early premature children has led to the identification of different subtypes of NEC, such as isolated perforations, that do not fit the classical description. Amongst the various risk factors for this pathology, prematurity represents the most common one [1]. A few studies have reported cases of NEC in full term neonates [3-5], with variable survival rates. For full term neonates, the reported incidence varies between one for every 20000 births and 10 percent of all NEC [6]. Studies have highlighted the frequency of underlying causes in this population [3-5, 7], such as congenital heart disease, congenital malformations (gastroschisis, Hirschsprung’s disease), neonatal hypoxemia, neonatal hyperglycemia and other maternal conditions.

We reviewed our experience with full-term neonates with NEC and to compare its characteristics to those published in the literature.

## MATERIALS AND METHODS

We reviewed all the data related to neonates managed for NEC, in the Reunion Island, between 2000 and 2012.

All patients born after 35 weeks of gestation, who met the specific criteria described below, were included [8]. The diagnosis of NEC required the presence of one or more of the following three clinical signs; (1) bilious gastric aspirate or emesis, (2) abdominal distension, (3) occult or gross blood in stool (without anal fissure) and one or more of the three following radiographic findings; (1) pneumatosis intestinalis, (2) hepato-biliary gas and (3) pneumoperitoneum. Focal gastrointestinal perforations were excluded from the study. We recorded all relevant maternal and infant details, including details of maternal illnesses during and prior pregnancy, mode of delivery, birth weight, gender, Apgar score at 5 minutes, date and type of feeding prior to disease onset, blood glucose levels, noticeable events prior to disease onset, other documented diseases, age at onset of first symptoms, results of the abdominal X-ray, blood count and culture results, duration of clinical and radiological disease, and early and late complications. All infants were explored by stool cultures for enterobacteria (Salmonella, Shigella, enteropathogenic Escherichia coli, Yersinia and Campylobacter) and viruses (enteroviruses and rota virus). Classical causes of NEC in full term infants, including Hirschsprung disease (by a systematic rectal biopsy), milk allergy, heart malformation or maternal or gestational disease, cystic fibrosis (by the assessment of trypsin blood level) were also searched for [4, 6]. 

When surgery was performed, timing of surgery was noted and per-operative observations were collected. Last follow up was also defined for each patient. 

We used Prism graphpad for analysis. Statistics are presented as mean +/-standard deviation.

## RESULTS

**Population**

Between 2000 and 2012, 6131 neonates were admitted in the Neonatal Intensive Care Unit in our institution. Among them, 217 (3.5%) were diagnosed having NEC. Only 27 (12.4%) were born after 35 weeks of gestation and met the diagnostic criteria for NEC.

Among them, there were 19 males and 8 females. Mean gestational age at birth (weeks) was 36.8 ±1.7. Modes of delivery included vaginal delivery in 19 and caesarean section in 8 (because of 3 abnormal foetal heart monitoring, 2 breeches, 2 abnormal presentations, 1 toxaemia). Mean birth weight was 2.6 kg ±0.7. Mean Apgar score at 5 minutes was 9.6±0.2. 

In 20 newborns, enteral feeding was initiated at a mean 1.2 ±0.8 days after birth with maternal milk for 14 and with artificial milk for the 6 others (and unknown in one). Among the 6 patients who remained with nil per os from birth, the causes were an early onset of the disease in 4 and the presence of cardiac malformations in 2 patients. Only 2 patients presented neonatal hypoglycaemia before the onset of NEC.

**NEC**

The disease began at a mean age of 12.1±11.2 days after birth. Three were previously discharged after birth and presented the initial symptoms of NEC at home, whereas the onset of the disease began at the hospital for the other 24 (88.8%). Hemodynamic status was defined as stable in 14 and unstable in 13 (requiring vasopressive medications). The biological work-up showed 230000±170000 platelets, a mean haemoglobin level of 12.2±4.2 g/dl and a C-Reactive Protein (CRP) level of 102±107 UI.

The mean duration of the antibiotic treatment was 9.9 ±7 days from the onset of the disease.

**Underlying causes**

Twenty patients had predisposing factors:

- Isolated maternal pathologies in 3 including gestational diabetes (n=2), and pregnancy induced hypertension (n=1).
- Infection in 2: congenital syphilis (n=1), Chikungunya infection both with maternal and foetal contamination (n=1). No CMV infection was found.
- Organic pathologies in 15 infants: 5 Hirschsprung’s diseases were discovered during the follow up, 4 cardiopathies (including one interventricular communication, 2 patent ductus arteriosus, 1 coarctation of the aorta), syndromic anorectal malformation (VACTERL), 3 cystic fibrosis, and 2 milk protein allergies. One patient developed NEC after meconium aspiration
- Seven patients developed idiopathic NEC. No NEC related pathology could be found, neither during neonatal screening nor infancy.

**Surgery**

NEC required surgery in 12 patients (37.5%) at a mean 23.2±20 days (Table 1). The delay between the onset of NEC and surgery was 15.8±19.3 days.

**Figure:**
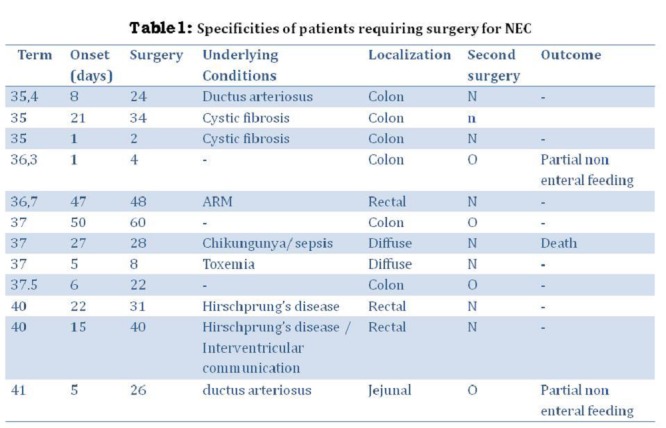
Table 1

Indications for surgery were:

- Pneumoperitoneum (n=5)
- Persistent occlusive symptomatology with blood in stools (n=2)
- Hemodynamic instability (n=1)
- Bacterial translocation with Hirschsprung’s disease (n=2) and
- Low anorectal malformation associated with oesophageal atresia (n=1)
- Klebsiella infection associated with cystic fibrosis (n=1)

The lesions involved the jejunum (1), multiple lesions of the small bowel (2), the colon (6) and the rectum in 3 according to peroperative observations.

The procedures consisted of:

Colostomy in 2 recto-sigmoid Hirschsprung’s disease and 1 anorectal malformation and 1 meconium ileus, which required diversion.

3 patients required complex procedures due to extensive colonic lesions. Advanced and diffuse lesions lead to ileal diversion. All patients underwent another procedure because of the occurrence of stenosis. In two cases, the stenosis was focal and localized on the left colon, whereas the last one required an extended colectomy because the lesions involved almost the entire colon.

**Outcome**

The overall survival rate was 88.8% (24/27). Three patients died during initial management due to the infection. Mean follow up was 3.7±1 years. The patient who underwent the extended colectomy required partial non-enteral nutrition for 1.3 years. A second patient required partial non-enteral nutrition for 2.1 years, because of an acute obstruction 2 months after the initial surgery, which required major bowel resection.

## DISCUSSION

Our study confirms that NEC in full term neonates remains a rare pathology with an incidence of 12.5% among all neonates with NEC. Most patients (81.5%) presented an underlying cause (maternal or intrinsic disease) including Hirschsprung’s disease and cardiac malformations. However in some cases, no evident cause was discovered. Among these patients, 3 developed clinical symptoms at home though they were considered healthy. The rest of the cohort developed NEC after being admitted in NICU for another pathology, which seems coherent with the current literature. Surgery was more likely to be necessary than in the premature population - 37.5% versus 22% (37/168).

The incidence of NEC in full term neonates has been evaluated at 1 per 20000 births in Australia [7], and these patients account for 7-10 % of all cases of NEC [6]. In our experience, 27 patients out of 217 who developed NEC were full term neonates (12.4%). 

No differences concerning presentation were found. Our survival rate (88%) is similar to reported previously. Lambert et al had found that the mortality rate of NEC is similar in both preterm and term neonates. [6]

As regards the timing of onset of the disease; contrary to most reports except the cohort reported by Lambert et al, the onset of NEC was slightly delayed in our series. Some authors [4] hypothesize that the early signs could be due to the consequences of a prenatal or an immediate neonatal ischemia, which triggers NEC. In our experience, only a few patients seemed to have experienced early neonatal injury that could have provoked the disease. 

The mode of delivery has been reported to be a potential cause of ischemic traumatism and has therefore been pointed out as a potential underlying cause [9]. In our experience, the mode of delivery and associated risk of birth asphyxia did not contribute to the onset of NEC and the timing of its occurrence. A low rate of birth asphyxia in both groups does not support this hypothesis as observed by Mayyan et al. [4]

The site of involvement of the gut was the colon or the rectum in about 75% of our cases. In the literature, similar to that reported by Mayyan et al [4] wherein 9 out of 14 patients developed colonic lesions. The pathogenesis of colonic involvement is not very clear except for the NEC secondary to ARM or Hirschsprung’s disease. Sepsis causing mesenteric vasoconstriction and ischemia or local bowel wall injury could be the underlying pathogenic mechanism in primary NEC. 

**Causes**

If prematurity remains the major risk factor for developing NEC, the presence of an underlying cause or event is classical in full term neonates [6]. The rate of patients with an identified predisposing factor varies from 50 to 100 percent in the literature [7, 10-12]. However, it depends on what was considered as a potential risk factor. Risk factors could be divided into 3 types:

- maternal and gestational conditions
- Organic pathology
- medical conditions that require neonatal management

In the first group, maternal diabetes, maternal drug abuses, preeclampsia, anti C Rhesus incompatibility, intrauterine growth retardation and premature rupture of the membranes have been recognized as potential causes (unknown physiopathology)[12]. 

In those with organic pathology, congenital cardiac anomalies seem to be a leading cause of NEC due to dimished mesenteric blood supply as stated by Mc Elhinney et al [13]. The frequency of these malformations ranges from 10-40% of full term neonates with NEC. In our series, only 4 patients presented with cardiac malformations. In this setting, the altered mesenteric flow easily explains the onset of the disease and the chronic and local hypoxemia is designated as the major cause for developing NEC [1]. Other surgical causes have also been reported such as gastroschisis or myelomeningocele [5, 14]. NEC represents a rare but classical complication of Hirschsprung’s disease or anorectal malformation, as confirmed in this series, and could be explained by local proliferation of bacteria in the bowel.

Finally, several medical conditions are listed as risk factors : hypoglycemia, polycythemia [15], cow’s milk allergy, or respiratory distress syndrome (causing general anoxia). As concerns an infectious origin, searching for enterobacteria is also mandatory during workup and several pathogens (including viruses) have been isolated in various epidemics. However, there was no evidence of such an epidemic in our experience. 

Chikungunya is an arbovirus virus transmitted by a mosquito (aedes albopictus), which can cause a disease with severe morbidity and mortality [16]. In 2005 and 2006, there was a large outbreak of Chikungunya in the Reunion Island, with more than 250000 cases declared. Materno-fetal infections were also observed [17] with one case of neonatal infection, complicated by NEC [18]. Transplacental transmission has been incriminated but the pathogenic mechanism remains unclear. In this case, NEC could be included as a consequence of the major septic syndrome due to the Chikungunia infection, which induced a diminished mesenteric flow. There was no evidence of a specific localisation of the virus, which could have suggested a direct insult.

Despite the wide range of etiologies, each study within the last decade has presented between 1 to8 patients without underlying cause [3, 5, 6], when minor medical conditions have been eliminated. In our experience, seven patients did not present any underlying cause or event in their medical background. Among them, 3 required emergency laparotomy with colonic stenosis in two and near total colonic stenosis in one. However, we found that each patient originated from the same region of our island, where ancestral customs are still active. We heard about enemas performed by this population during the neonatal period, which could explain the late onset of the disease, at home in 3 cases. But, without evidence, it is still a hypothesis. 

As regards feeding, it has been well demonstrated that breast milk feeding protects against NEC [1]. However, the frequency of breast feeding was equivalent to formula feeds in our experience, and brest feeding did not seem to be protective in the occurrence of NEC as seen in 3 of the full term neonates with colonic lesions who were all breast fed. This led us to look for other causes such as the ancestral customs mentioned earlier.

Recently, Lambert et al [6] concluded that artificial feeding was also a risk factor for developing NEC, along with a diminution of the mesenteric flow. In their study, 97% of patients were fed by formula, whereas previous studies reported various rate from 68% to 100%. Our observations seem different, but from the literature, it is clear that breast milk feeding is a protective factor against NEC even in full term neonates [2].

There were several limitations in our study. Most are the same as in previous reports and include the size of the cohort and the duration of the study. However, some conclusions can be drawn, which mainly confirm previous reports. First of all, NEC in full term neonates is a rare pathology, which concerned children with general diseases in most cases. The onset of the disease in our experience is slightly later than in the literature, but remains earlier than in the premature population. In some cases, no obvious cause can be found, suggesting a different pathogenesis. Further investigations are required in order to better understand this pathology. The goal will be to find measures to reduce global mortality. 

## Footnotes

**Source of Support:** Nil

**Conflict of Interest:** None

